# The Impact of Ultra-Marathon Running on the Gut Microbiota as Determined by Faecal Bacterial Profiling, and Its Relationship with Exercise-Associated Gastrointestinal Symptoms: An Exploratory Investigation

**DOI:** 10.3390/nu17203275

**Published:** 2025-10-18

**Authors:** Kayla Henningsen, Stephanie K. Gaskell, Pascale Young, Alice Mika, Rebekah Henry, Ricardo J. S. Costa

**Affiliations:** 1 Department of Nutrition, Dietetics and Food, Sports and Exercise Dietetics & Extremes Physiology Research Group, Monash University, Notting Hill, VIC 3168, Australia; kayla.henningsen@monash.edu (K.H.); stephanie.gaskell@monash.edu (S.K.G.); pascale.young@monash.edu (P.Y.); alicesmika@gmail.com (A.M.); 2 School of Public Health and Preventive Medicine, Monash University, Clayton, VIC 3168, Australia; rebekah.henry@monash.edu

**Keywords:** ultra-endurance, microbiota, EIGS, exercise-associated gastrointestinal symptoms

## Abstract

**Background/Objectives:** This exploratory study aimed to evaluate the impact of an 80 km ultra-marathon trail running event on changes in faecal bacterial composition, and to investigate whether any correlations exist between exercise-associated gastrointestinal symptoms (Ex-GIS) with faecal bacterial profiles. Such events represent a unique physiological stressor and may impact the composition of the gut microbiota. Studying this impact may provide insights into acute (i.e., <24 h) gut microbiota changes under extreme conditions. **Methods:** Thirteen endurance athletes (*n* = 7 males, *n* = 6 females) aged 41 ± 8 years completed the 80 km Margaret River (Australia) ultra-marathon race in 2022. Faecal samples were collected pre- and post-race. Faecal bacterial profile, as per relative abundance (RA) of operational taxonomic units and the determination of α-diversity (Shannon Equitability Index (SEI)), was achieved by 16S rRNA amplicon gene sequencing. Changes in RA% and SEI pre- to post-race were assessed by the Wilcoxon signed-rank test. Correlations between Ex-GIS with bacterial profile and changes pre-, during, and post-ultra-marathon race were determined by Spearman’s rank correlation coefficients. **Results:** Bacterial calculations of phyla (*n* = 5), family (*n* = 23), and genus (*n* = 41) were detected for RA (≥0.5%). A significant decrease pre- to post-race of *Actinobacteriota* (*p* = 0.035) phyla, *Bifidobacteriaceae* (*p* = 0.007), and *Clostridiaceae* (*p* = 0.010) family, and *Blautia* (*p* = 0.039) and *Subdoligranulum* (*p* = 0.023) genus was determined; meanwhile, *Oscillospiraceae* (*p* = 0.016) and *Monoglobaceae* (*p* = 0.039) family significantly increased pre- to post-race. No other bacterial group changes were observed. No correlations were observed between pre- to post-ultra-marathon RA change and Ex-GIS. **Conclusions:** The completion of an 80 km ultra-marathon did not invoke substantial changes in the gut microbiota as determined by faecal bacterial profiling. Very strong and strong correlations were observed between certain bacterial groups and Ex-GIS; however, no significant correlations were observed between pre- to post-ultra-marathon changes in RA ≥ 0.5% and Ex-GIS.

## 1. Introduction

Ultra-endurance sport is growing in popularity, with increasing participation across events such as single- and multi-stage ultra-marathons, ultra-distance triathlons, adventure racing, open-water swimming, and other activities lasting ≥ 4 h [1,2]. These events impose significant physiological demands due to prolonged exertion, often compounded by environmental stressors like extreme temperatures (≤0 °C to ≥30 °C), high altitudes (>3000 m), and rugged terrain [1]. Among these, ultra-marathon running (defined as any race exceeding 42.195 km) has seen notable growth in participation and adherence [3,4,5]. A key clinical concern with rising ultra-marathon participation is increased susceptibility to exercise-induced gastrointestinal syndrome (EIGS), which arises from two primary mechanisms [6]. First, prolonged exertion redirects blood flow away from the gastrointestinal tract, causing splanchnic hypoperfusion and intestinal epithelial damage, leading to increased permeability and impaired absorption [6,7,8,9,10,11]. Second, neuroendocrine disturbances, including elevated cortisol and sympathetic drive, suppress gut motility via altered enteric nervous system activity [7]. These pathways may facilitate translocation of luminal pathogens into systemic circulation, potentially triggering systemic inflammation and severe outcomes such as sepsis or multi-organ failure [7,12,13,14,15,16,17,18,19,20,21]. Various intrinsic and extrinsic factors may exacerbate EIGS onset [7,22,23].

One intrinsic factor warranting further investigation is the role of the gut microbiota in modulating EIGS and the severity of exercise-associated gastrointestinal symptoms (Ex-GIS) [7]. The gut bacterial profile may exert protective or harmful effects depending on its composition [19,22,24,25,26,27,28]. Increased α-diversity and relative abundance (RA) of commensal bacteria (e.g., *Lachnospiraceae*, *Ruminococcus*, and/or *Bacteroides*) are associated with production of short-chain fatty acids (SCFAs) and other metabolites that enhance gut immunity, epithelial integrity, and motility [19]. Conversely, prolonged exertion may alter gut pH during hypoperfusion, disrupting bacterial function and potentially converting commensal bacteria into opportunistic pathogens [19,23]. These microbiota-mediated changes may contribute to the onset and severity of Ex-GIS, which frequently accompany EIGS. Over 60% of ultra-endurance athletes have reported symptoms such as nausea, bloating, and diarrhoea [6,12,29]. Upper-GIS symptoms are more common in dehydrated athletes or those exercising in heat, while lower-GIS symptoms like flatulence and bloating are often linked to prolonged activity (>4 h) [12,29]. Severe systemic EIGS has been associated with symptoms including vomiting and/or bloody defecation, likely due to compromised gut integrity and immune responses [1,9,10,12,30,31,32,33].

Currently, there is no standardised profile of the athlete’s gut microbiome, with significant intra- and inter-individual variability reported [22,24,25,32]. Literature on acute (<24 h) microbiome changes in response to exercise remains limited. Given the extremes of ultra-endurance events, it is plausible that changes to the bacterial profile of the gut microbiota would be substantially pronounced with ultra-endurance event participation. It also remains unclear whether these changes correlate with Ex-GIS severity. Accordingly, the aims of this study were to (1) determine whether completing an 80 km ultra-marathon trail event alters gut bacterial composition via faecal profiling; and (3) evaluate whether these changes correlate with Ex-GIS onset. We hypothesised that participation in an 80 km ultra-marathon trail running event would result in measurable alterations to gut bacterial composition, specifically (1) a reduction in Shannon Equitability Index (SEI) and relative abundance (RA) of short-chain fatty acid (SCFA)-producing commensal bacteria (e.g., *Lachnospiraceae*, *Ruminococcus*, and/or *Bacteroides*); and (2) an increase in RA of potentially pathogenic or opportunistic bacteria. Furthermore, we hypothesised that these microbiota changes would be positively correlated with the incidence and severity of exercise-associated gastrointestinal symptoms (Ex-GIS), as reported by the participants post-race.

## 2. Materials and Methods

### 2.1. Participants

Thirteen endurance-trained athletes [mean ± SD (*n* = 7 males, *n* = 6 females): age 41 ± 8 years, height 174 ± 9 cm, body mass 66.5 ± 7.7 kg, weekly training load 456 ± 158 min/week] provided written informed consent to volunteer to participate in the study. The study protocol received approval from the local ethics committee (Monash University Human Research Ethics Committee (MUHREC: 29429) and conformed to the 2024 Helsinki Declaration for Human Research Ethics. The inclusion criteria for this study were endurance-trained athletes aged between 18 and 50 years who were free of illness and/or disease. All participants freely and voluntarily entered the Margaret River ultra-marathon. Participants were excluded from the study if they had gastrointestinal infections, diseases and/or disorders (e.g., coeliac disease, inflammatory bowel disease, irritable bowel syndrome, diverticular disease, gastro-esophageal reflux disease, history of gastrointestinal surgery, and/or other self-reported gastrointestinal ailments), consumed potential modifiers of gastrointestinal integrity (e.g., prebiotics, probiotics, and/or antibiotics), adhered to gastrointestinal-focused dietary regimes (e.g., low fermentable oligo, di-, mono-saccharide, and polyol (FODMAP) and/or fibre modified diets) within the previous 3 months, or consumed nonsteroidal anti-inflammatory medications and/or stool altering medications (e.g., laxatives and antidiarrheal) within 1 month before the ultra-marathon race.

### 2.2. Experimental Procedures

**Figure 1:**
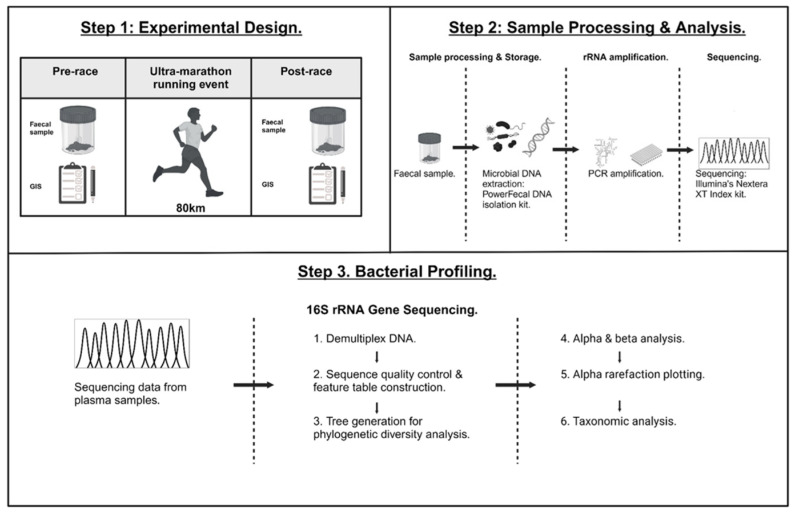
Schematic illustration of the experimental, sample processing, and analysis procedures.

Experimental procedures are demonstrated in Figure 1. To minimise participant burden on race day, baseline characteristics and resting biomarker data were obtained during the afternoon prior to the ultra-marathon race. All participants completed the 80 km ultra-marathon race, with Ex-GIS data recorded via recall of the entirety of the race by the athletes. Ex-GIS was determined via an exercise-specific, validated, and reliability-checked modified visual analogue scale (mVAS) gastrointestinal symptoms assessment tool [31]. The rating scale of the mVAS GIS tool is as follows: 0 indicates no GIS experienced, 1–4 indicates mild GIS (i.e., the athlete is aware of the symptoms; however, the symptoms are not severe enough to impact exercise workload and/or cessation from the activity), 5–9 indicates severe GIS (i.e., GIS is severe enough to noticeably impact exercise workload, but not to the point of cessation of the activity), and 10 indicates extreme GIS and cessation from the activity [31]. A comprehensive dietary assessment and analysis was collected 72 h prior to the ultra-marathon, in accordance with the method described by Costa et al., 2014 [34]. Total energy (kCal), protein, carbohydrate, fat, water, and dietary fibre consumption were quantified via FoodWorks dietary analysis software (FoodWorks 10 Professional, v10.0. Brisbane, Australia: Xyris Pty Ltd., 2019).

### 2.3. Faecal Sample Collection and Bacterial Profiling

All participants (*n* = 13) were instructed to provide an approximately 30 g mid-flow faecal sample into a sterile collection container (SARSTEDT Australia Pty Ltd., Mawson Lakes, South Australia, Australia), which was promptly stored at −20 °C and subsequently transferred to −80 °C for long-term preservation until further processing and analysis. All post-race measures were repeated in the same sequence within 15–30 min of race completion to ensure procedural consistency. Competitors were given a 24-hour window to submit their initial post-race faecal sample. Faecal samples were thawed to room temperature and homogenised, allowing for 0.20–0.30 g of each sample to be transferred into a 2 mL dry garnet bead microtube, in addition to bead solution. Mechanical and chemical cell lysis, sample purification, and DNA extraction were then completed according to the manufacturer’s instructions (PowerFecal DNA isolation kit, Qiagen, Germantown, TN, USA), with pyrogen/DNAse/RNAse-free consumables within a biohazard ventilation cabinet to avoid artefact contamination (Safemate 1.2 ECO, AF Technologies Pty Ltd., Baywater North, Victoria, Australia). Blank control samples (pyrogen/DNAse/RNAse-free water) were run simultaneously in duplicate and demonstrated minimal presence of microbial DNA concentration after analysis (11,730 and 11,065). Similarly, positive control samples (ZymoBIOMICS Faecal Reference with TruMatrixTM Technology) were also run simultaneously in duplicate and demonstrated vast amounts of microbial DNA concentration (5,458,933 and 5,099,250). An aliquot of 50 µL of the purified extracted DNA sample was immediately frozen at −20 °C prior to bacterial gene sequencing. The extracted genomic DNA was delivered to Micromon Genomics (Monash University, Clayton, Australia) for PCR amplification of the V3-V4 region of the 16S rRNA gene and sequencing. Reactions contained 1 μM forward (TCGTCGGCAGCGTCAGATGTGTATAAGAGACAGCCTAC GGGNGGCWGCAG) and reverse (GTCTCGTGGGCTCGGAGATGTGTATA AGAGACAGGACTACHVGGGTATCTAATCC) primers; 5 μL of purified concentration-standardised genomic DNA; 25 μL of 2× HiFi HotStart ReadyMix (Kapa Biosystems, Wilmington, MA, USA). Amplified DNA, derived from the triplicate reactions, was then pooled for each sample and purified using Ampure Axygen AxyPrep PCR Clean Mag Beads XP (0.6 V) according to the manufacturer’s instructions. The libraries were then pooled in equimolar concentrations and sequenced using a MiSeq V3 600c Reagent Kit (Illumina, San Diego, CA, USA). The assembled reads were then analysed via QIIME2 (v.2019.1) software, and underwent quality assessment, filtering, barcode trimming, and chimaera detection via the DADA2 pipeline [22]. Taxonomic evaluation was achieved with a 98% identity and confidence value of *p* ≤ 0.05% with the SILVA 138.1 release. Sequence variance counts for phyla, family, and genus were determined by dividing the number of reads of each taxon by the total number of reads from each faecal sample. 16S rRNA sequences per faecal sample ranged from 7924 to 20,140,332, and a rarefaction sampling depth of 197,298 was utilised for analysis due to the high sequencing yield observed across the majority of samples, allowing for consistent comparison of diversity metrics without excluding any samples from analysis. For amplicon sequence variants (AVS), only bacterial groups with ≥ 0.5% RA across the combined dataset (i.e., pooled pre- and post-ultra-marathon samples) were included for data analysis to avoid the risk of the inclusion of artefact values in data analysis, resulting from potential contamination during sample handling (i.e., sample collection, processing, and analysis). Bacterial calculations of phyla (5 taxa), family (23 taxa), and genus (41 taxa) were adequately detected for RA (≥0.5%) and SEI determination.

### 2.4. Statistical Analysis

Given the exploratory nature of the present study, the statistical power was deemed sufficient based on prior laboratory research demonstrating exercise-induced disturbances in gastrointestinal integrity, systemic responses, and changes in faecal bacterial profiles [12,29]; nevertheless, post hoc statistical power analysis was performed on G*Power (version 3.1), and relevant effect sizes were reported. Statistical analysis was completed with the use of SPSS statistical software (V.29.0, Chicago, IL, USA) with significance accepted at *p* ≤ 0.05. Descriptive data within the text are demonstrated as mean ± SD. Data within the tables are demonstrated as SEI, RA (%) of total identified bacteria, or mean ± SD. Comparison data of faecal bacterial composition pre- to post-ultra-marathon was examined by using the Wilcoxon signed-rank test. Spearman’s Bivariate Correlation coefficient was used to determine potential correlations between Ex-GIS and bacterial group RA changes pre- to post-ultra-marathon.

## 3. Results

### 3.1. Dietary Intake

Inter-individual variability of total energy (1420–3773 kCal/day), protein (52–193 g/day), carbohydrate (23–543 g/day), fat (34–181 g/day), water (698–4218 mL/day), and dietary fibre (11–60 g/day) consumption for 72 h prior to the ultra-marathon event was demonstrated among the athletes that recorded their dietary intake. Given the sample size and the descriptive nature of the dietary intake data, these findings should be interpreted with caution and not overemphasised; they primarily serve to illustrate the extent of inter-individual variability in nutritional strategies prior to the ultra-marathon.

### 3.2. Faecal Microbial Taxa

The faecal bacterial taxa SEI and RA of predominant phyla (5 taxa), family (23 taxa), and genus (41 taxa) groups are demonstrated in Table 1. Before the ultra-marathon race, the identification of RA of bacterial phyla taxa in faecal samples included *Firmicutes*, *Bacteroidota*, *Actinobacteroita*, *Verrucomicrobiota*, and *Proteobacteria* (Supplementary Figure S1). The identification of the relative abundance of bacterial family taxa was also achieved. The predominant bacterial family taxa were *Lachnospiraceae*, *Ruminococcaceae*, *Bacteroidaceae*, *Bifidobacteriaceae*, *Peptostreptococcaceae*, *Akkermansiaceae*, *Prevotellaceae*, *Coriobacteriaceae*, *Erysipelatoclostridiaceae*, *Oscillospiraceae*, *Streptococcaceae*, *Clostridiaceae*, *Christensenellaceae*, *Rikenellaceae*, *Eggerthellaceae*, *Tannerellaceae*, *Barnesiellaceae*, *Erysipelotrichaceae*, *Coprostanoligenes*, *Enterobacteriaceae*, *Monoglobaceae*, *Butyricicoccaceae*, and *Veillonellaceae* (Supplementary Figure S2). Similarly, the identification of the relative abundance of bacterial genera was also sufficient. The predominant bacterial genera were *Blautia*, *Bacteroides*, *Faecalibacterium*, *Bifidobacterium*, *Agathobacter*, *Subdoligranulum*, *Ruminococcus*, *Anaerostipes*, *Akkermansia*, *Fusicatenibacter*, *Eubacterium hallii group*, *Collinsella*, *Coprococcus*, *Dorea*, *Prevotella*, *Erysipelotrichaceae UCG-003*, *Clostridium*, *Streptococcus*, *Christensenellaceae*, *Romboutsia*, *Alistipes*, *Parabacteroides*, *Ruminococcus torques group*, *Barnesiella*, *Intestinibacter*, *CAG-352*, *Eubacterium Coprostanoligenes*, *Escherichia-Shigella*, *UCG-002*, *Roseburia*, *Monoglobus*, *Holdemanella*, *Ruminococcus gauvreauii group*, *Incertae Sedis*, *NK4A214 group*, *Lachnospiraceae*, *Butyricicoccus*, *Lachnospiraceae ND3007*, *Adlercreutzia*, *Phascolarctobacterium*, and *Dialister* (Supplementary Figure S3). As demonstrated in Table 1, there was no significant difference (*p* > 0.05) in phyla, family, and/or genus SEI pre- to post-ultra-marathon. However, a significant alteration in bacterial RA for phyla, family, and genus groups pre- to post-ultra-marathon did occur (e.g., *Actinobacteriota* phyla (−1.372%), *Bifidobacteriaceae* family (−0.258%), and *Subdoligranulum* genus (−0.959%)).

### 3.3. Exercise-Associated Gastrointestinal Symptoms (Ex-GIS)

Ex-GIS reported pre-, during, and immediately post-ultra-marathon race is presented in Table 2. All participants reported some form of Ex-GIS along the ultra-marathon. Ex-GIS types reported pre-, during, and post-ultra-marathon were, however, mild in severity (mVAS < 5). The most commonly reported upper-GIS reported was belching, whilst flatulence was the most commonly reported lower-GIS. Ex-GIS incidence pre-ultra-marathon accounted for 11.7% of the mean summative score of symptom incidence. Symptoms reported during the ultra-marathon accounted for 61.5% of the mean summative score of symptom incidence. Meanwhile, symptoms reported post-ultra-marathon account for 27.5% of the mean summative score of symptom incidence.

### 3.4. Bacterial RA and Ex-GIS

**Figure 2:**
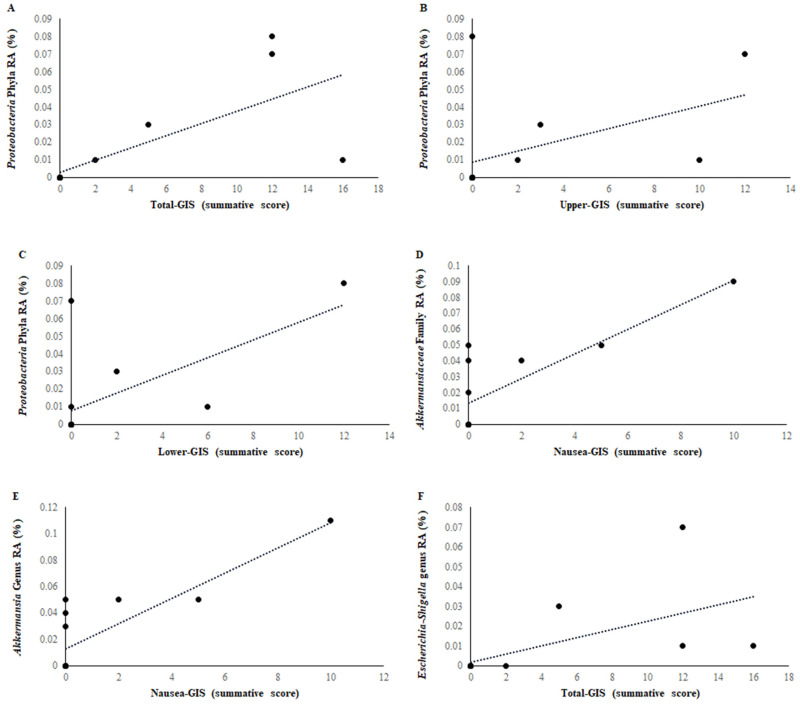
Post-ultra-marathon bacterial RA (%) correlation with Ex-GIS: (**A**): (*Proteobacteria* phyla RA and total-GIS), (**B**): (*Proteobacteria* phyla RA and upper-GIS), (**C**): (*Proteobacteria* phyla RA and lower-GIS), (**D**): (*Akkermansiaceae* family and nausea-GIS), (**E**): (*Akkermansia* genus and nausea-GIS), and (**F**): (*Escherichia-Shigella* genus and total-GIS).

Several statistically significant positive and negative correlations were observed between bacterial RA change pre- to post-ultra-marathon and Ex-GIS occurrence for phyla, family, and genus bacterial groups. A very strong positive correlation between *Proteobacteria* phyla and total-GIS (r = 0.838, *n* = 12, *p* < 0.001) was demonstrated pre- to post-ultra-marathon (Figure 2A). Strong positive correlations pre- to post-ultra-marathon were demonstrated between *Proteobacteria* phyla (r = 0.643, *n* = 12, *p* = 0.018) and lower-GIS, *Proteobacteria* phyla (r = 0.638, *n* = 12, *p* = 0.019) and upper-GIS, *Akkermansiaceae* (r = 0.721, *n* = 12, *p* = 0.008) family, and *Akkermansia* (r = 0.721, *n* = 12, *p* = 0.008) genus and nausea-GIS, and *Escherichia-Shigella* (r = 0.663, *n* = 12, *p* = 0.013) genus and total-GIS (Figure 2B–F). Moderate positive correlations were demonstrated between *Christensenellaceae* (r = 0.587, *n* = 12, *p* = 0.035) and *Escherichia-Shigella* (r = 0.568, *n* = 12, *p* = 0.043) genus and upper-GIS. Meanwhile, *Phascolarctobacterium* (r = 0.554, *n* = 12, *p* = 0.049) and *Escherichia-Shigella* (r = 0.573, *n* = 12, *p* = 0.041) genus had moderate positive correlations with lower-GIS. Strong negative correlations were demonstrated between *Firmicutes* (r = −0.606, *n* = 12, *p* = 0.028) phyla and *Rikenellaceae* (r = −0.629, *n* = 12, *p* = 0.028) family and lower-GIS. Strong negative correlations were also demonstrated between *Firmicutes* (r = −0.611, *n* = 12, *p* = 0.027) phyla and *Streptococcaceae* (r = −0.624, *n* = 12, *p* = 0.030) family and upper-GIS, *Firmicutes* (r = −0.684, *n* = 12, *p* = 0.010) phyla and *Rikenellaceae* (r = −0.738, *n* = 12, *p* = 0.006) family and total-GIS, and *Veillonellaceae* (r = 0.611, *n* = 12, *p* = 0.014) family, *Lachnospiraceae ND3007* (r = −0.635, *n* = 12, *p* = 0.020) genus and nausea-GIS. However, when limiting the analysis to taxa with a relative abundance of ≥ 0.5% that also showed a significant pre- to post-race change in RA, none of these taxa were significantly correlated with Ex-GIS.

## 4. Discussion

The aims of the present study were to determine whether completing an 80 km ultra-marathon trial running event impacts the gut bacterial composition, as determined by faecal bacterial profiling, and to investigate whether changes in bacterial composition are correlated with Ex-GIS occurrence, which are known to develop as a consequence of EIGS. Contrary to our hypothesis, the current study did not establish a uniform ‘athlete gut microbiota’, instead showing large intra- and inter-individual variation within a relatively homogeneous population of endurance and ultra-endurance recreational athletes. These findings differ from previously reported faecal bacterial profile data in similar ultra-endurance cohorts [19,22,24]. Instead, the findings revealed substantial diversity in gut bacterial composition among ultra-endurance athletes, with completion of the 80 km ultra-marathon not leading to significant changes in faecal SEI. However, notable alterations were observed in the RA of specific bacterial phyla, families, and genera in response to the ultra-endurance race. Furthermore, no bacterial taxa that had a significant RA% change pre- to post-ultra-marathon were found to be correlated with the onset of Ex-GIS. Nevertheless, correlations between certain bacterial groups and specific Ex-GIS were observed. These findings suggest that individual gut bacterial responses to ultra-marathon trail running are highly variable, highlighting the need for personalised approaches to managing Ex-GIS in athletes, as the gut microbiota changes and/or ultra-marathon-induced changes appear not to contribute primarily to the Ex-GIS. Moreover, considerable variability in dietary intake among participants in the 72 h prior to the event may have further contributed to the heterogeneity in faecal microbial taxa and should be carefully considered in future research investigating gut-related responses in endurance athletes [22,24]. Additionally, while significant structural changes in gut microbiota composition are demonstrated after hours to days of gut microbiota-altering stimuli, the current study aimed to capture gut microbiota dynamics immediately following an extreme physiological stressor. Thus, a post-race sampling window of 15–30 min was utilised to not imply full community restructuring, but rather to explore potential transient microbial responses that may reflect early shifts in overall gut microbiota composition in response to the ultra-marathon stressor. Emerging evidence supports the plausibility of rapid microbial responses to stimuli. David et al. (2014) [35] have demonstrated that dramatic dietary changes can evoke detectable alterations in gut microbial gene expression within hours. Similarly, Zhao et al. (2018) [36] report that acute endurance exercise affects gut microbial activity shortly after exertion, underlying the gut microbiota’s sensitivity to acute perturbations. In light of the intensity and duration of the 80 km ultra-marathon event, it is biologically plausible that early gut microbiota responses may be detectable within minutes post-race. However, it is acknowledged that stable compositional shifts that indicate structural changes in gut microbiota composition require longer durations for significant detection to be achieved (i.e., 24 h following a stimulus, reflecting immune modulation, dietary intake, and circadian rhythms). Sampling at 15–30 min post-race offers a unique opportunity to isolate immediate exertion-induced gut microbiota shifts, and may minimise confounding influences that may emerge during the recovery phase. Thus, the findings of the current study contribute to a growing body of research investigating acute shifts in gut microbiota dynamics. The significance of optimised gut health, particularly for athletes, is well-documented in the current literature [6,37]. Maintaining gut health in athletes is critical for preserving enterocyte integrity, mucosal barrier function, and tight-junction protein stability and function; thereby, preventing the translocation of pathogenic agents from the gut lumen into systemic circulation in response to exertional activity may contribute to altered thermoregulatory responses, i.e., fever associated with systemic inflammatory responses [14,33,38], and lead to clinically significant and irreversible outcomes [6]. Previous studies have investigated the impact of exercise on the composition of gut bacterial phyla, families, and genera [14]. This research has identified the predominant bacterial phyla, families, and genera in faecal samples before and after exercise. Specifically, *Firmicutes*, *Bacteroidota*, *Actinobacteria*, *Proteobacteria*, and *Verrucomicrobia* have been consistently observed as the dominant phyla pre-exercise [24,25]. For the family bacterial taxa, *Ruminococcaceae*, *Lachnospiraceae*, *Bacteroidaceae*, *Acidaminococcaceae*, *Prevotellaceae*, *Christensenellaceae*, *Veillonellaceae*, *Rikenellaceae, Muribaculaceae*, *Akkermansiaceae*, *Pasteurellaceae*, and *Bifidobacteriaceae* are typically predominant in faecal samples before exercise [24,25]. Similarly, common bacterial genera found pre-exercise include *Bacteroides*, *Faecalibacterium*, *Agathobacter*, *Phascolarctobacterium*, *Prevotella*, *Blautia*, *Christensenella*, *Roseburia*, *Subdoligranulum*, *Alistipes*, *Veillonella*, and *Eubacterium* [24,25]. In alignment with these prior studies, the current study also identified a similar predominant bacterial phyla composition (Table 1). However, in contrast to previous research, the present study revealed variations in the most dominant bacterial family and genus groups among the participants (Table 1). This is not surprising, considering nearly all studies that have analysed athlete faecal samples to establish bacterial profiles are not consistent with each other [14]. The differences in bacterial composition of the current study highlight the individualised nature of gastroenterology research and underscore the inherent challenges of studying gut microbiota in athletic populations, and thus, identifying an ‘athlete microbiome’ [7].

As previously discussed, the concept of an ‘athlete microbiome’ is widely accepted. However, the hypothesis that all athletes share a uniform gut bacterial profile is misleading and warrants further clarification. While it is well established that regular physical activity positively influences (e.g., increases α-diversity) the gut microbiota of athletes compared to sedentary individuals [23,37,39,40,41,42,43,44,45,46], the notion of a universal gut bacterial profile among athletes is virtually unattainable [47,48]. As demonstrated in Table 1, a variety of commensal bacterial phyla, families, and genera significantly increased and decreased in response to the ultra-marathon race, thereby confounding the notion that an “athlete’s microbiome”, particularly post-exercise, is only composed of beneficial bacteria proliferation. Research into the gut microbiota is inherently constrained by several confounding factors that extend beyond methodological control. Firstly, it is important to note that a 10% variability in gut bacterial profiles exists among individuals, regardless of their physical activity levels [49]. This variation can be attributed to factors such as differences in faecal water concentration and motility time among individuals [49]. Moreover, studies assessing the gut microbiota of athletes commonly rely on faecal samples, which is the case of the current study, and represent bacterial composition from the colon. This poses a limitation, as the true depth of microbial activity occurs along the entire gastrointestinal tract, in which the small intestine (e.g., terminal ileum) is abundant with microbial activity [50,51]. This distinction is especially relevant when examining the impact of the gut microbiota on the incidence of EIGS. Additionally, techniques such as culturomics and metagenomic assays, which are used to characterise microbial profiles, fail to distinguish between live and dead bacteria. Dead bacteria, along with bacterial metabolites such as secondary bile acids, SCFAs, protein derivatives, and other unidentified bacterial metabolites, are collectively referred to as ‘dark matter’. This ‘dark matter’ significantly influences the gut microbiota composition, often beyond the scope of methodological control [52,53,54]. Finally, it is anticipated that pH alterations within the gut lumen, induced by prolonged exertional activities, such as ultra-marathons, may further impact the gut bacterial profile of athletes. These pH shifts can result from factors such as splanchnic hypoperfusion, mechanical stress, and/or altered gastric emptying [19]. Changes in pH can either promote the growth or induce the death of certain bacterial species, complicating the accurate profiling of the gut microbiota. Collectively, these factors generate a highly variable and uncontrollable environment, significantly impeding efforts to accurately characterise an individual’s gut bacterial profile and further complicating the task of profiling such microbiota within a cohort of athletes. Ultimately, the concept of a uniform ‘athlete microbiome’ is oversimplified, as individual variability, methodological limitations, and physiological factors create a complex and dynamic microbial landscape that challenges accurate characterisation in athletic populations.

### 4.1. Exercise-Associated Gastrointestinal Symptoms

As previously discussed, alterations in gut microbiota composition have been implicated in the incidence of EIGS. However, it remains unclear whether these changes also contribute to the incidence of Ex-GIS. The onset of Ex-GIS typically occurs once EIGS has already been established within the affected athlete. Once symptoms emerge, degradation of gastrointestinal enterocyte integrity, potential translocation of pathogenic agents from the gut lumen into systemic circulation, and an inflammatory response have already transpired. Thus, it is difficult to decipher if the Ex-GIS onset is due to potential gut microbiota changes from exertional activity or occurs in response to prolonged EIGS.

In the current study, several statistically significant correlations were observed between changes in bacterial RA from pre- to post-ultra-marathon and Ex-GIS occurrence. Notably, a very strong positive correlation was demonstrated between *Proteobacteria* phylum and total-GIS (r = 0.838, *n* = 12, *p* < 0.001), with additional strong positive correlations between *Proteobacteria* and both upper- and lower-GIS, *Akkermansiaceae* family and *Akkermansia* genus with nausea-GIS (r = 0.721, *n* = 12, *p* = 0.008) and *Escherichia-Shigella* with lower-GIS. Conversely, strong negative correlations were found between *Firmicutes* phylum and lower-, upper-, and total-GIS, as well as between *Rikenellaceae* and *Streptococcaceae* families and various GIS. The *Lachnospiraceae ND3007* genus also demonstrated a strong negative correlation with nausea-GIS. However, when the analysis was restricted to taxa that both (1) had a relative abundance ≥ 0.5% and (2) showed a statistically significant change from pre- to post-race, none of these taxa were significantly correlated with Ex-GIS. This suggests that while several exploratory associations were observed, the onset of Ex-GIS may not solely be dependent on immediate gut microbiota shifts induced by ultra-endurance activity. Rather, it may reflect a more complex relationship involving chronic microbial patterns shaped by lifestyle, diet, illness, and/or medication use. These findings highlight the need for further mechanistic and longitudinal studies to clarify the role of the gut microbiota in Ex-GIS pathophysiology.

### 4.2. Impact of Bacterial Microbial Composition on EIGS and Ex-GIS

Understanding how changes in gut microbial composition influence the pathophysiology of EIGS and the subsequent onset of Ex-GIS remains a critical area of investigation. A diverse and balanced gut microbiota, particularly one with a high RA of beneficial commensal bacteria, plays a central role in maintaining gut health. These bacteria contribute to gut integrity and host defence by producing SCFAs such as butyrate, acetate, and propionate, as well as other anti-inflammatory metabolites [19,55]. These compounds support gut function by enhancing luminal immunity (e.g., stimulating antimicrobial protein secretion and activating innate immune responses), strengthening the epithelial barrier (e.g., increasing mucus production, enterocyte proliferation, and tight junction expression), inhibiting the adhesion of pathogenic bacteria to the intestinal lining, and promoting healthy gut motility [19,33,56,57]. However, prolonged exertional activity, such as that experienced during ultra-endurance events, can introduce several physiological stressors, including splanchnic hypoperfusion, mechanical strain, and delayed gastric emptying [19,33,58]. These factors may lower gut luminal pH, which in turn can negatively impact the gut microbiota by disrupting the balance between commensal, pathogenic, and opportunistic bacteria [19,33]. Such alterations may exacerbate or accelerate the onset of EIGS, creating a cyclical relationship between microbiota disruption and gastrointestinal injury that ultimately contributes to the development of Ex-GIS. When EIGS becomes well established, particularly in the context of a dysregulated gut bacterial microbial profile, the integrity of the gastrointestinal barrier may become compromised, allowing bacterial endotoxins and/or whole bacteria to translocate into systemic circulation [14,33,59]. This can provoke a systemic inflammatory response, which, if left unmanaged, may progress to serious clinical outcomes such as sepsis, multiple organ failure, or death [14,33,57,60]. From a practical perspective, these findings underscore the importance of supporting gut microbial health in endurance athletes. Maintaining a gut microbiota that is rich in commensal bacteria may offer protective effects against the onset and severity of EIGS and Ex-GIS, especially during periods of prolonged exertional stress.

This study provides important preliminary insights into gut microbiota responses to ultra-marathon participation under real-world conditions. As with any exploratory research, several limitations should be considered. Dietary intake was not standardised or controlled, in order to reflect the natural variability of ultra-endurance settings, but this limited our ability to fully isolate exercise effects from nutritional influences. Diet is a well-established modulator of gut microbiota composition [22]. In the context of ultra-endurance events, athletes often consume high-carbohydrate, high-sugar, and/or specialised nutritional supplements before and during the race. Thus, dietary fluctuations may have introduced inter-individual variability that may have masked or diluted true exercise-induced gut microbiota shifts, particularly for bacterial taxa that may be sensitive to macronutrient consumption and/or fermentable substrates.

The modest sample size (*n* = 13), while sufficient for detecting primary outcomes, constrained the statistical power for more nuanced analyses, such as β-diversity and correlation assessments. As this is the first study employing this novel methodology (gastrointestinal microbiota shifts in response to a multi-stage marathon in ambient conditions), there were no prior data available to support a meaningful a priori power calculation. However, from the observed effect sizes of a post hoc power analysis, the authors recommend a minimum sample size of *n* = 35 in future studies to achieve sufficient power for detecting statistically significant changes. This recommendation is intended to guide future research building on this novel approach. Additionally, while the authors prioritised identifying potential associations, formal corrections for multiple comparisons were not applied, and some significant findings had small effect sizes. These results should therefore be interpreted as hypothesis-generating rather than confirmatory. Correlations with gastrointestinal symptoms, particularly where compositional changes were not observed, highlight the complexity of host–microbiota interactions and the need for functional validation. Overall, these limitations are typical of early-phase, field-based microbiota research, and the study provides a strong foundation for future investigations using larger cohorts, controlled dietary protocols, functional and biomarker integration, and mechanistic validation.

## 5. Conclusions

In conclusion, this exploratory study sheds light on the nuanced relationship between ultra-endurance running and gut microbiome dynamics. Although the overall bacterial SEI remained stable following the 80 km ultra-marathon, distinct shifts in the relative abundance of specific microbial taxa were evident. Notably, several bacterial groups demonstrated significant correlations with Ex-GIS, including strong positive associations between *Proteobacteria*, *Akkermansia*, and *Escherichia-Shigella* and various GIS such as total-, upper-, lower-, and nausea-related symptoms. Conversely, *Firmicutes*, *Rikenellaceae*, and *Streptococcaceae* showed strong negative correlations with Ex-GIS, suggesting a potential protective role. However, when analyses were restricted to taxa that both significantly changed pre- to post-race and had an RA ≥ 0.5%, no significant correlations with Ex-GIS were observed. This suggests that acute microbial fluctuations may not be the primary drivers of Ex-GIS, and that observed associations may reflect broader, possibly chronic, microbial patterns or host responses. These findings underscore the complexity of host-microbiome interactions during extreme physical exertion and highlight specific bacterial groups that may warrant further investigation as potential contributors to Ex-GIS. Further research should aim to validate these associations in larger cohorts and explore mechanistic pathways linking gut microbiota to GIS in ultra-endurance athletes.

## Figures and Tables

**Table 1:** Shannon Equitability Index (SEI) and relative abundance (RA%) of faecal bacterial phylum, family, and genus AVS at rest prior to and post an 80 km ultra-marathon trail running event.

	Pre-Ultra-Marathon	Post-Ultra-Marathon	∆
Faecal phylum			
SEI	0.171 (0.035)	0.179 (0.032)	0.008 (0.034)
*Actinobacteriota*	8.808 (4.432)	7.436 (5.896)	−1.372 (4.210) *
*Bacteroidota*	17.119 (10.753)	21.234 (9.285)	−4.115 (9.533)
*Firmicutes*	70.364 (8.152)	67.523 (7.607)	−2.841 (7.895)
*Proteobacteria*	0.878 (2.231)	1.604 (2.532)	0.726 (2.244)
*Verrucomicrobia*	2.827 (3.312)	2.200 (2.754)	−0.627 (2.765)
Faecal family			
SEI	0.234 (0.032)	0.233 (0.024)	−0.001 (0.039)
*Bifidobacteriaceae*	5.073 (3.862)	4.815 (4.708)	−0.258 (1.699) *
*Coriobacteriaceae*	2.592 (2.409)	1.994 (1.663)	−0.597 (0.763)
*Bacteroidaceae*	10.490 (8.573)	11.933 (7.631)	1.443 (3.171)
*Prevotellaceae*	2.696 (4.307)	5.122 (8.546)	2.425 (2.653)
*Erysipelotrichaceae*	1.021 (1.901)	1.113 (2.513)	0.091 (0.849)
*Lachnospiraceae*	36.141 (10.366)	34.510 (9.168)	−5.8543 (3.405)
*Oscillospiraceae*	2.356 (1.860)	2.771 (2.196)	0.414 (0.789) *
*Ruminococcaceae*	18.790 (5.770)	19.943 (7.182)	1.152 (2.589)
*Peptostreptococcaceae*	2.937 (4.154)	1.352 (1.619)	−1.584 (0.900)
*Akkermansiaceae*	2.927 (3.404)	2.294 (2.897)	−0.633 (1.264)
*Eggerthellaceae*	1.072 (1.020)	0.684 (0.585)	−0.388 (0.290)
*Barnesiellaceae*	1.035 (1.124)	1.144 (1.066)	0.109 (0.431)
*Rikenellaceae*	1.264 (1.319)	1.494 (1.390)	0.229 (0.541)
*Tannerellaceae*	1.067 (1.210)	1.324 (1.072)	0.257 (0.452)
*Erysipelatoclostridiaceae*	2.495 (2.828)	2.225 (2.965)	−0.270 (1.158)
*Streptococcaceae*	1.635 (3.402)	1.166 (1.226)	−0.468 (1.032)
*Christensenellaceae*	1.552 (1.881)	1.262 (1.496)	−0.289 (0.655)
*Clostridiaceae*	1.581 (4.236)	0.577 (1.270)	−1.003 (0.417) *
*Monoglobaceae*	0.736 (0.612)	0.741 (0.728)	0.005 (0.265) *
*Butyricicoccaceae*	0.642 (0.805)	0.526 (0.416)	−0.115 (0.258)
*Coprostanoligenes*	0.797 (0.674)	0.717 (0.476)	−0.080 (0.207)
*Veillonellaceae*	0.312 (0.547)	0.860 (1.557)	0.548 (0.446)
*Enterobacteriaceae*	0.776 (2.351)	1.419 (2.647)	0.642 (1.001)
Faecal genus			
SEI	0.265 (0.021)	0.264 (0.015)	0.001 (0.017)
*Bifidobacterium*	5.406 (4.039)	5.144 (4.993)	−0.262 (1.781)
*Bacteroides*	11.422 (9.543)	12.852 (8.296)	1.430 (3.507)
*Agathobacter*	4.718 (3.835)	5.431 (4.568)	0.713 (1.654)
*Anaerostipes*	3.188 (2.514)	3.492 (3.210)	0.304 (1.131)
*Blautia*	13.810 (7.621)	10.543 (4.801)	−3.267 (2.498) *
*Fusicatenibacter*	2.923 (1.908)	3.088 (2.641)	0.164 (0.903)
*Eubacterium hallii group*	2.890 (1.808)	3.465 (2.054)	0.575 (0.759)
*Faecalibacterium*	11.224 (7.208)	12.114 (5.194)	0.890 (2.464)
*Ruminococcus*	3.206 (2.626)	3.574 (2.915)	0.368 (1.088)
*Subdoligranulum*	3.720 (1.434)	22.761 (1.427)	−0.959 (0.561) *
*Collinsella*	2.807 (2.621)	2.120 (1.731)	−0.686 (0.871)
*Adlercreutzia*	0.351 (0.565)	0.230 (0.298)	−0.121 (0.177)
*Barnesiella*	1.090 (1.194)	1.204 (1.150)	0.113 (0.113)
*Prevotella*	1.909 (3.724)	4.681 (9.559)	2.772 (2.845)
*Alistipes*	1.251 (1.363)	1.487 (1.545)	0.235 (0.571)
*Parabacteroides*	1.160 (1.336)	1.413 (1.134)	0.253 (0.486)
*Erysipelotrichaceae UCG-003*	1.798 (2.650)	1.561 (3.024)	−0.236 (1.115)
*Holdemanella*	0.782 (1.936)	0.862 (2.655)	0.079 (0.911)
*Streptococcus*	1.719 (3.549)	1.218 (1.262)	−0.501 (1.044)
*Christensenellaceae*	1.704 (2.121)	1.385 (1.701)	−0.318 (0.754)
*Clostridium*	1.780 (4.856)	0.650 (1.479)	−1.129 (1.408)
*Lachnospiraceae*	0.715 (0.605)	0.877 (0.666)	0.162 (0.249)
*Coprococcus*	2.129 (1.676)	2.002 (1.420)	−0.126 (0.609)
*Dorea*	2.094 (0.966)	2.143 (1.035)	0.048 (0.392)
*Lachnospiraceae ND3007*	0.606 (1.066)	0.700 (1.019)	0.093 (0.409)
*Roseburia*	0.786 (0.723)	0.979 (0.969)	0.192 (0.335)
*Ruminococcus gauvreauii group*	0.754 (0.909)	0.682 (0.882)	−0.072 (0.351)
*Ruminococcus torques group*	1.140 (0.930)	1.017 (1.035)	−0.122 (0.386)
*Monoglobus*	0.784 (0.636)	0.791 (0.762)	0.007 (0.275)
*Butyricicoccus*	0.667 (0.863)	0.529 (0.437)	−0.139 (0.268)
*NK4A214 group*	0.740 (0.944)	0.868 (1.132)	0.128 (0.409)
*UCG-002*	0.801 (0.899)	0.916 (0.922)	0.115 (0.357)
*CAG-352*	0.906 (2.785)	0.752 (2.620)	−0.154 (1.060)
*Incertae Sedis*	0.753 (0.900)	1.792 (5.083)	1.039 (1.431)
*Eubacterium Coprostanoligenes*	0.865 (0.735)	0.777 (0.522)	−0.088 (0.250)
*Intestinibacter*	1.071 (2.035)	0.523 (0.705)	−0.548 (0.597)
*Romboutsia*	1.665 (3.400)	0.666 (0.848)	−0.999 (0.972)
*Phascolarctobacterium*	0.347 (0.421)	0.502 (0.498)	0.154 (0.181)
*Dialister*	0.322 (0.591)	0.715 (1.333)	0.393 (0.404)
*Escherichia-Shigella*	0.811 (2.446)	0.954 (1.975)	0.143 (0.872)
*Akkermansia*	3.155 (3.662)	2.514 (3.286)	−0.641 (1.364)

Mean (SD) of ≥0.5% RA (*n* = 13): * *p* < 0.05 pre- to post-ultra-marathon. AVS: amplicon sequence variant, RA: relative abundance, SEI: Shannon Equitability Index.

**Table 2:** Incidence and severity of GIS in response to an 80 km ultra-marathon trail running event.

	Ex-GIS Incidence (%)	Ex-GIS (Severity ^#^)
Total-GIS	100	8 (2-48)
Upper-GIS	92	4 (1-27)
Belching	92	1 (1-5)
Heartburn (gastro-oesophageal reflux)	31	1 (1-8)
Upper abdominal bloating	46	1 (1-8)
Upper abdominal pain	31	0 (1-4)
Urge to regurgitate	38	1 (1-10)
Regurgitation	8	0 (10-10)
Projectile vomiting	15	1 (10-10)
Lower-GIS	92	4 (1-25)
Flatulence	77	2 (1-7)
Lower abdominal bloating	54	1 (1-7)
Urge to defecate	54	1 (3-10)
Left intestinal cramps	15	0 (3-4)
Right intestinal cramps	23	0 (2-5)
Defecation (loose stools)	8	0 (10-10)
Defecation (diarrhoea)	0.00	0 (0-0)
Defecation (bloody stools)	0.00	0 (0-0)
Nausea	46	1 (1-10)
Dizziness	38	1 (1-9)
Stitch (acute transient abdominal pain)	38	1 (1-8)

Ex-GIS = exercise-associated gastrointestinal symptoms. ^#^ Mean of summative accumulation and range of participants reporting incidence (*n* = 13).

## Data Availability

The data presented in this study are available upon reasonable request from the corresponding author. The data are not publicly available due to privacy and confidentiality of data in accordance with the ethics committee-approved procedures.

## Supplementary Materials

The following supporting information can be downloaded at: https://www.mdpi.com/article/10.3390/nu17203275/s1, Figure S1: RA% of bacterial phyla taxa in faecal samples collected pre- and post-ultra-marathon from *n* = 13 participants.; Figure S2: RA% of bacterial family taxa in faecal samples collected pre- and post-ultra-marathon from *n* = 13 participants.; Figure S3: RA% of bacterial genus taxa in faecal samples collected pre- and post-ultra-marathon from *n* = 13 participants.
