# Supplementary material for: The Impact of Ultra-Marathon Running on the Gut Microbiota as Determined by Faecal Bacterial Profiling, and Its Relationship with Exercise-Associated Gastrointestinal Symptoms: An Exploratory Investigation
Source: Nutrients. 2025 Oct 18;17(20):3275. doi: 10.3390/nu17203275 (PMC12567543; doi:10.3390/nu17203275)
Supplement: Supplementary file 1 [file nutrients-17-03275-s001.zip › nutrients-3749057-supplementary.pdf]

Supplementary Materials

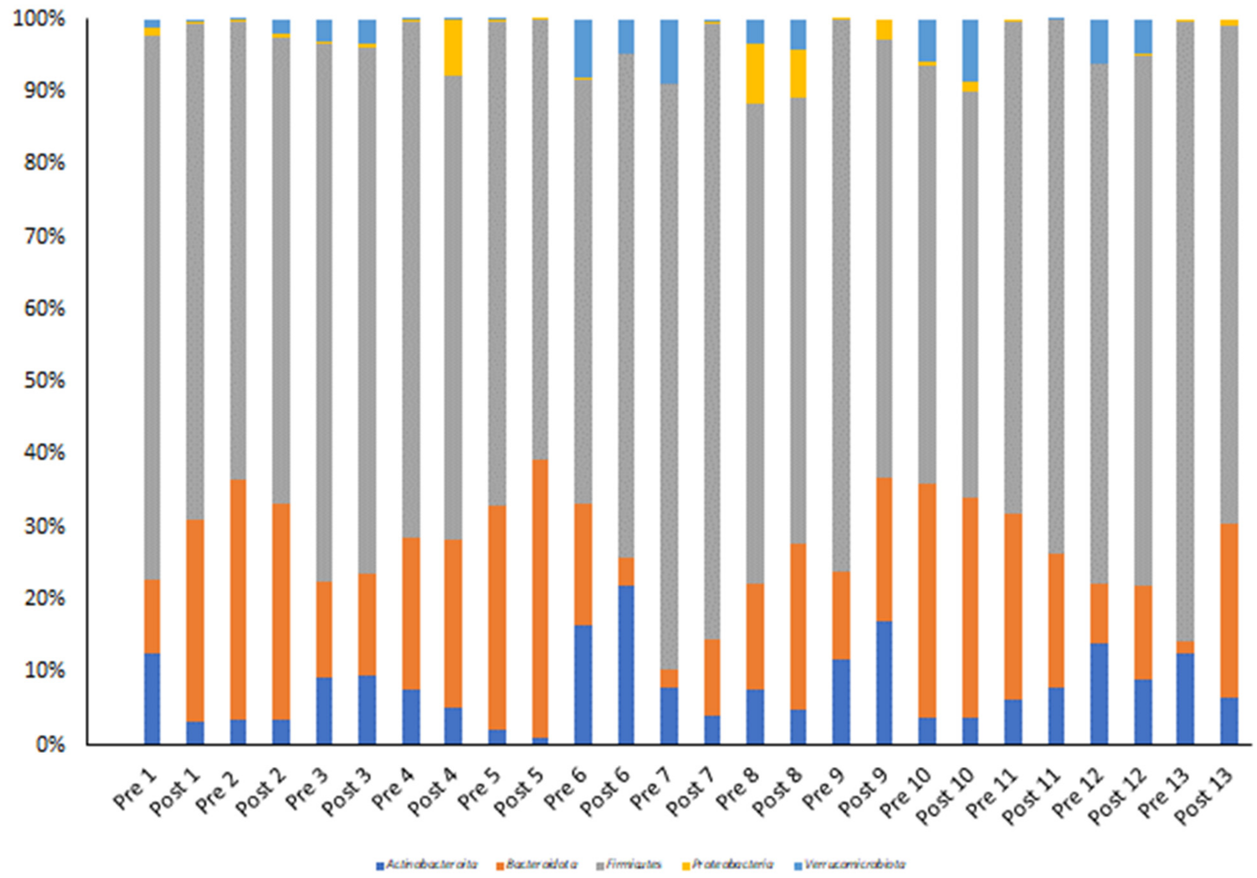

Supplementary Figure S1. RA% of bacterial phyla taxa in faecal samples collected pre- and post-ultra-marathon from n = 13 participants.

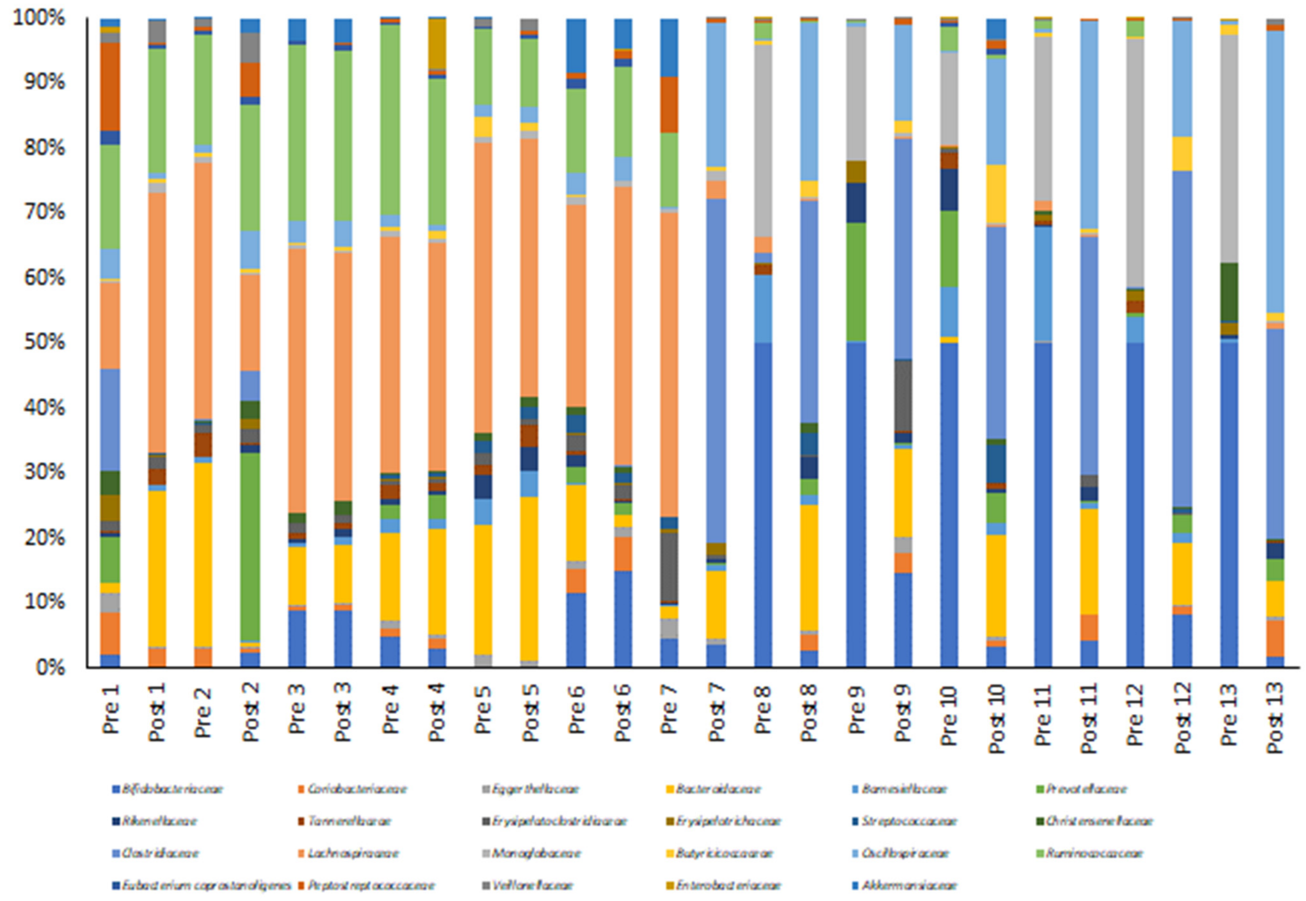

Supplementary Figure S2. RA% of bacterial family taxa in faecal samples collected pre- and post-ultra-marathon from n = 13 participants.

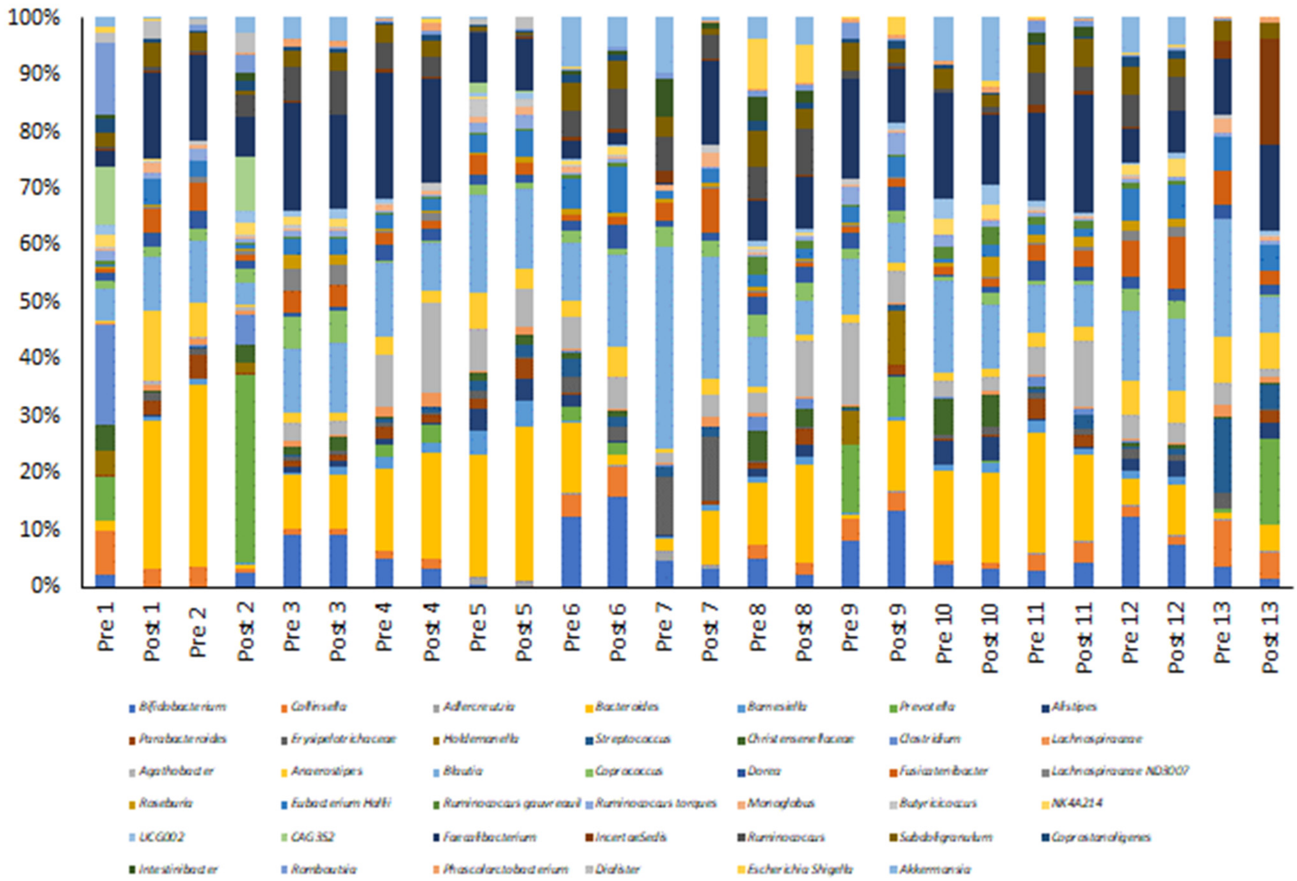

Supplementary Figure S3. RA% of bacterial genus taxa in faecal samples collected pre- and post-ultra-marathon from n = 13 participants.
